# Churn prediction of mobile and online casual games using play log data

**DOI:** 10.1371/journal.pone.0180735

**Published:** 2017-07-05

**Authors:** Seungwook Kim, Daeyoung Choi, Eunjung Lee, Wonjong Rhee

**Affiliations:** Department of Transdisciplinary Studies, Seoul National University, Seoul, Republic of Korea; East China University of Science and Technology, CHINA

## Abstract

Internet-connected devices, especially mobile devices such as smartphones, have become widely accessible in the past decade. Interaction with such devices has evolved into frequent and short-duration usage, and this phenomenon has resulted in a pervasive popularity of casual games in the game sector. On the other hand, development of casual games has become easier than ever as a result of the advancement of development tools. With the resulting fierce competition, now both acquisition and retention of users are the prime concerns in the field. In this study, we focus on churn prediction of mobile and online casual games. While churn prediction and analysis can provide important insights and action cues on retention, its application using play log data has been primitive or very limited in the casual game area. Most of the existing methods cannot be applied to casual games because casual game players tend to churn very quickly and they do not pay periodic subscription fees. Therefore, we focus on the *new* players and formally define churn using observation period (*OP*) and churn prediction period (*CP*). Using the definition, we develop a standard churn analysis process for casual games. We cover essential topics such as pre-processing of raw data, feature engineering including feature analysis, churn prediction modeling using traditional machine learning algorithms (logistic regression, gradient boosting, and random forests) and two deep learning algorithms (CNN and LSTM), and sensitivity analysis for *OP* and *CP*. Play log data of three different casual games are considered by analyzing a total of 193,443 unique player records and 10,874,958 play log records. While the analysis results provide useful insights, the overall results indicate that a small number of well-chosen features used as performance metrics might be sufficient for making important action decisions and that *OP* and *CP* should be properly chosen depending on the analysis goal.

## Introduction

The global game market continues to grow, and it is expected to grow 9.6 percent per year between 2013 and 2018 with a stable revenue stream [1]. As the number of published games increase exponentially, game market is becoming too complicated to understand as a single sector. Therefore, researchers often divide the market into multiple sectors and analyze each sector separately. One way of categorizing games is by checking the type of platform: console, handheld, personal computer, online, smartphone, tablet, or television [1]. Another way is to base the categorization on the complexity and intensity. According to [2], hardcore games are the traditional and complex ones that require players to spend substantial time and resources, and *casual games*, such as Tetris, Pac-man, and Wii, are the simple ones that are easy to play and require little time and few resources. According to [3], casual games can be characterized by short and less complex gameplay sessions, easiness for learning and playing, and platforms that are ordinary devices like personal computers and mobile phones. According to [4], casual games are ‘normalization of digital play’ and have interfaces that use only few buttons. While it is difficult, if not impossible, to unambiguously define casual games in a concise way, obviously casual games have become very popular with a high growth rate since 2000 [5] and they are the primary subject of this study.

With the number of smartphone users rising, the mobile game market has been an important part of the business. In the mobile market, casual games are one of the three most popular game types, and there are many famous casual games such as ‘Angry birds (2010),’ ‘Fruit Ninja (2010),’ and ‘Candy crush (2012)’ [6]. Many casual games use *freemium*, a pricing strategy that provides products for free but with in-app purchases. Especially in most iOS and Android market categories, freemium is an effective pricing strategy and is used very often. Approximately ninety percent of revenue in games comes from in-app purchases in free apps [7]. The success of those games relies on in-game purchases and in-game advertising to generate revenue. In other words, the more users there are, the more revenue game suppliers generate.

When a new casual game is released, the company focuses on marketing to attract new users. However, if the number of users is sufficient, the company would do better by focusing more on user retention, because a small change in the retention rate can have much larger impacts on the profit. For instance, 5% growth in retention rate resulted in a roughly four times larger profit according to [8]. The key to retaining users is to identify who will or will not be out of service. This is well-known to be possible, because typically users who will be out of service appear to differ [9]. We use the term *churn* to refer to the circumstance that someone who was in a service leaves it; such a person is called a churner. Churn analysis or prediction defines who will or will not churn, and the churn rate is the ratio of churners to non-churners during a specific time period. In other words, suppliers need to lower the churn rate of their users [10]. Typically, churn can be utilized in two different ways. First, churn rate can be evaluated as a business metric such that the status of business can be understood and monitored. For casual games, an example is to monitor churn rate of a newly released game and to estimate if the game is likely to be successful or not. Often a good indication leads to an increased marketing budget. Second, churn prediction can be applied to individual users such that a targeted action can be taken to the users who are likely to churn or are deemed to be important. For casual games, an example is to provide free items or to send marketing messages to ‘likely-to-churn’ users.

User churn prediction is crucial in the game industry, in which low churn rates are directly connected to revenue stability. The application of behavior analytics, viz. churn analysis, has emerged from the sidelines to become a core component of commercial and academic game development, especially during the past five years [11]. Under the influence of this phenomenon, many analysis services for game developers have also emerged, and companies have worked to retain users. Compared to the changes and importance in the industry, however, it is quite difficult to find published research results on game and its churn analysis, especially for casual games. For sophisticated games, such as MMORPG (massively multiplayer online role-playing games), research results are available for churn causes including bots and cheating [12][13], account theft [14], and cyberbullying [15][16].

There are a few aspects to consider when studying churn of casual games. For telecommunication or newspaper industries, the business is based on subscription. Therefore, a churner can be very clearly defined when the subscriber notifies termination of service. Some of the sophisticated games, such as MMORPG, are based on subscription as well. Casual games, however, are widely based on freemium models where players might not return to the game for a few months or even for good without notifying anyone or paying monthly charges. For such dormant players, it becomes impossible to have one single golden definition of churn, and an adequate definition needs to be adopted in accordance to the main analysis purpose—one might be interested in if a player will return in 10 days in one case, and 1 month in another case. In this study, a flexible churn definition using observation period (*OP*) and churn prediction period (*CP*) is provided to address this issue.

Another aspect to consider is the target group to analyze. While most of the existing churn analyses focus on the entire user base, another important approach is to consider the new users only. In most of the industries, churn rate is much higher for the newly acquired group as the users try out the service. Therefore, it becomes crucial to fully understand the new users and address their issues. For casual games, this effect is pronounced even more. For the three casual games that we analyze in this study, 70~90% of the players stop playing within 10 days from the first day of play. Apparently new player analysis is essential for monitoring and improving the casual games.

In this study, we focus on new players of non-subscription-based casual games, and establish a standard procedure of analysis. Three datasets are used, where two are mobile casual games and one is online casual game. We build an analysis model by observing a new player’s play log data from the first *OP* days of play and predicting if the player will play again in the following *CP* days. Note that a player might not play for the *CP* days but then return at a time in a farther future. Such a player is still considered as a churner in this study, because otherwise it is impossible to define ‘ground truth’. To explain this, assume that we make *CP* infinite and eliminate the ‘returning at a farther future’ problem. Then, no matter how long we wait, we cannot tell if a player is really a churner or not. The player is not paying subscription fee, and therefore there will be no special action such as a service termination notice. To avoid this problem, we need to compromise, set *CP* at a reasonable value, and make it possible to identify churners by looking into *CP* days of future. This turns out to be a practical compromise, because most of the players who do not return in *CP* days do not return at least until the end of data collection period that is way much later than *CP* days.

In the following, we summarize the related works, formally define churn of casual games, and explain the data. Then, feature engineering is performed over three games to understand the data details and the influence of common and game-specific features. Performance results follow, where prediction performance for five different prediction algorithms is compared and sensitivity to the different selections of *OP* and *CP* is investigated. AUC (area under the curve) of ROC (receiver operating characteristic) [17][18] through ten-fold cross-validation [19] is used as the performance measure. Overall, we derive interesting insights on mobile and online casual games through the churn prediction analysis.

## Related works

Early user churn prediction research has been conducted in a variety of commercial fields such as telecommunication, newspaper, bank, credit card, and insurance [20] [21] [22] [23]. In each field, churn prediction has evolved in accordance to the industry development.

As the game industry has matured, a number of user churn prediction studies have been conducted because the insights from such studies can have critical influences on the game revenue. Runge et al. (2014) performed a churn prediction research for high-value players in casual social games, and provided a framework for game churn analysis [24]. They predicted churn for the target group of top 10% of high paying users of two freemium mobile casual games using four algorithm types (neural network, logistic regression, decision tree, and support vector machine), and defined a churner to be a player with no play for 14 consecutive days. The model performance was evaluated using AUC of ROC, and the effectiveness of prediction model was gauged using A/B test. While [24] focused on high-value players who show long lifetimes, this work focuses on casual players who typically show very short lifetimes after the first play of a game.

Hadiji et al. (2014) explained the definition of user churn prediction as two concepts and formulated the input data for the prediction as two methods as well [11]. They used decision tree, logistic regression, neural network, and naïve Bayes algorithm models, i.e., four models using freemium game data for each of four input data sets generated according to the two definitions of user churn and the two methods. F1-score was used to compare the models’ performance, and the decision tree model performed best.

Kawale et al. (2009) determined the factor that affects user churn in two ways, social influence among users and their personal engagement in MMORPGs [25]. The social influence factor was used to create a network graph using group-play records. The personal engagement factor was used to create a beta function using user session time records. Furthermore, they created a modified diffusion model based on an existing simple diffusion model for evaluating performance and described model-dependent processes that classify users who will or will not churn.

Another noticeable change in the research is the use of deep neural networks (DNN). DNN have improved remarkably on some traditional research problems, such as image classification and speech recognition [26][27][28]. Wangperawong et al. (2016) applied deep CNNs and autoencoders to telecommunication churn analysis and showed that their approach outperforms decision tree modeling with 12 temporal features for each user [29].

Although the importance of user churn prediction of games has been acknowledged and research results including the aforementioned ones are available, our understanding on churn analysis of games is far from being complete or mature. Overall, the existing works seem to have mainly three limitations, and our study focuses on the three. First, most studies focus on rather blindly defining features for predicting user churn and do not provide in-depth analysis. Prediction performance significantly varies depending on how we define features, and it is difficult to make churn rate improvement without fully understanding the chosen features and their implications in the domain [30][31][32]. Thus, we provide the details of defining and analyzing features in this study. Second, algorithms as well as features for a prediction model can have a substantial effect on building a prediction model, but there have been limitations to using algorithms in past studies [33]. Hence, we design prediction models using three representative algorithms together with two of the latest deep learning models, and compare the performances of those models. Third, existing studies set the observation period (*OP*) and churn prediction period (*CP*) arbitrarily without conducting additional studies for setting those periods. However, the definition of the churn is directly dependent on the two periods and the prediction results and performance can substantially depend on them. To overcome this limitation, we analyze their effects and provide useful insights.

## Churn definition

We focus on the new players of casual games (no subscription). To study churn, we predict if a new player will stop playing after a certain number of days since the first day of play. As addressed in the introduction, this definition of churn is quite different from the case of targeting ‘the entire user base’ where the question is if an existing user, regardless of the length of in-service period, will still pay in the following month. To formally define a churner for casual games, we first introduce a few notations. For a player *u*, let *s*_*i*_ and $t_{i}^{abs}$ represent the score and timestamp, respectively, of the player’s *i*^*th*^ play. Because we focus on new players only, it is convenient to define $t_{i}^{rel}$, relative time with respect to the player’s first play time. For the simplicity of the notation, we use *t*_*i*_ to represent $t_{i}^{rel}$ in the rest of the paper.

$$t_{i}=\{\begin{array}{ccc} 0 & , & i=1\\ t_{i}^{abs}-t_{1}^{abs} & , & i\neq 1 \end{array}$$

As an example, if the timestamp of a player’s first play is 1,469,623,182 and the timestamp of the third play is 1,469,633,182, then *t*_3_ is 10,000 seconds. Note that *t*_1_ = 0 by definition, and it is emphasized in the above equation.

**Fig 1:**
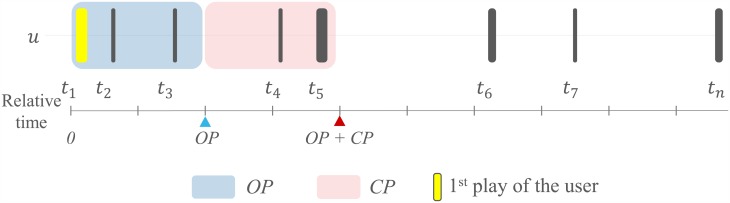
Time diagram of a player’s play log data. The player has played *n = 8* times. The first three plays occurred within two days of the first play. The next two plays occurred in the following two days.

We define a churner in terms of observation period (*OP*) and churn prediction period (*CP*). An *OP* is a period for observing a user’s plays, and the play log data are used to create features and predict churn for the following *CP* days. A *CP* is a period for determining whether a user actually churned or not. Using the two periods, a churner is defined as a user playing the game for the first time when *t*_1_ = 0 and possibly more in the first *OP* days, and not playing the game at all in the following *CP* days. The definition is for each individual player, and the player’s own clock since the first play time is used for defining *OP* and *CP*. Fig 1 shows a time-diagram example. Together with the 8 plays that the user has played, observation period of 2 days (*OP* = 2) and churn prediction period of 2 days (*CP* = 2) are highlighted in the diagram. In reality, larger *OP* and *CP* values are preferred, but small values are shown here to simplify the diagram. As explained in the introduction, the play activity after the *OP+CP* does not affect if the user is a churner or not. Unlike subscription-based services, this is necessary for freemium casual games because we cannot wait forever for deciding if a user is a churner or not. A decision needs to be made within a reasonable time frame to allow a meaningful analysis and action for the new users. Despite of the limitation, this definition turns out to be quite reasonable and practical, because most of the churners indeed do not return to the game forever when *CP* is larger than just several days. This is due to the nature of casual games where the players are not serious.

**Fig 2:**
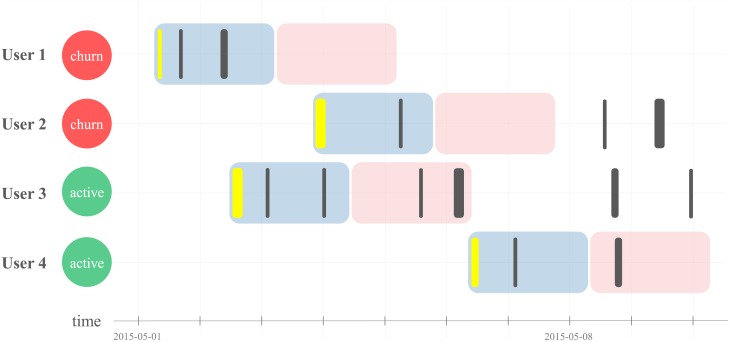
Two churners and two active users. Each row describes play log records of a user, and a user with no play log record in the churn prediction period is defined as a churner. When desired, a larger *CP* can be chosen for defining churn.

In Fig 2, two examples of churners and two examples of active users are shown. User 1 has three play records in *OP* but no play record in *CP*, and therefore User 1 is a churner. User 2 has active play records after *OP+CP* days, but it is also a churner according to our definition. Again, this type of player is seldom found in reality compared to the type of User 1. In contrast, if a user has play records in the *CP*, the user is called an active user. User 3 has three play records in the *OP* and two records in the *CP*, and User 4 has 2 play records in *OP* and 1 play record in *CP*.

For the freemium games, the choice of *OP* and *CP* needs to be made prudently depending on the goal of churn analysis. If a quick and targeted action is needed within the first 3 days of a player’s first play, obviously *OP* needs to be set at 3 days or below. On the other hand, if a popularity metric for a newly released game is needed, *OP* can be set to a larger number such that the prediction accuracy can be enhanced. For *CP*, one could choose a small value to predict the short term behaviors or choose a large value to predict the long term behaviors. In the rest of this paper, we fix *OP* to 5 days and *CP* to 10 days and perform feature engineering and algorithm performance analysis. The studies were completed for many different *OP* and *CP* choices as well, but the main insights remain the same and hence we fix the values and avoid over-complications. Instead, we provide explicit analysis results on the impact of *OP* and *CP* choices in the ‘Performance for different choices of *OP* and *CP*’ section.

## Data

The three casual games chosen for churn prediction are Dodge the Mud, a casual racing game with an undisclosed title (per data owner’s request), and TagPro. Dodge the Mud is a casual action game on Android. In the game, a player dodges mud coming down from the sky by touching buttons on the phone screen or tilting the phone; the player receives scores, which are proportional to the number of muds the player dodges. Game #2 is a casual racing game on Android and iOS. A player rides a vehicle over challenging terrain with a specified time limit. Then, the player receives game money, which can be used to upgrade the vehicle. Lastly, TagPro is a casual CTF (capture-the-flag) game on a web browser. It is a team game in which a player seizes the other team’s flag and brings it back to his or her own team. The player then receives individual play scores and team points, which can be earned only when the player’s team wins a game. Table 1 summarizes the three games, with data descriptions of each game.

**Table 1:** Three games and their data.

Game	Game #1	Game #2	Game #3
**Title**	Dodge the Mud	Undisclosed	TagPro
**Genre**	Casual action	Casual racing	Casual CTF
**Platform**	Android	Android, iOS	Web Browser
**Data collection period**	2015-01-15 ~ 2016-05-24	2014-06-16 ~ 2014-10-01	2015-05-26 ~ 2015-12-25
**Number of log records**	153,876	7,620,127	3,100,955
**Number of users**	25,954	79,436	88,043

The raw play log records of Game #1 were available in MySQL database. The raw data of Game #2 was provided in text files with the contents in a JSON (JavaScript Object Notation) format. For Game #3, a public API for downloading play log data was available, and JSON files were obtained through the API. From the raw datasets, redundant, irrelevant, and not-so-informative data fields were filtered out after a careful examination and pre-processing. The resulting data fields that are used in this research are summarized in Tables 2–4. Among the three games, Game #2 is the only game where items can be purchased. As will be discussed in the feature engineering section, **purchase.price** is used for game-specific feature extractions. For Game #3, the **play.duration** and **win** are used for game-specific feature extractions. The last attribute, **points**, is different from **score** because the accumulated value of **points** is used to decide rankings, and subsequently the list of players to list in the leaderboard. While the high-rankers can be motivated to continue playing, this attribute was not considered in this research because typically only 100 players are listed in the leaderboard.

**Table 2:** Principal attributes of Game #1.

Attribute	Description
**device.id**	Unique device id assigned by smartphone manufacturer (used as unique user id)
**time**	End time of the play
**score**	Score of the play

**Table 3:** Principal attributes of Game #2.

Attribute	Description
**device.id**	Unique device id assigned by smartphone manufacturer (used as unique user id)
**time**	End time of the event
**event**	Type of this record (play or purchase)
**score**	Score of the play (when **event** is play)
**purchase.price**	Purchase price (when **event** is purchase)

**Table 4:** Principal attributes of Game #3.

Attribute	Description
**id**	Unique user ID (log-in ID)
**play.id**	Unique ID of the play
**time**	End time of the play
**play.duration**	Duration of the play
**win**	Team’s win or loss
**score**	Score of the play
**points**	Points earned from the play (accumulated points are used to rank players and announce top leaders in a leaderboard)

## Feature engineering

For traditional machine learning algorithms to work well, an appropriate feature engineering needs to be performed. When done properly, feature engineering not only improves the algorithm performance but also enables discovery of important insights. In this section, intuitively meaningful features are extracted first, and their importance is evaluated by analyzing single feature ranking and by identifying the best feature combinations.

### Feature extraction

Feature extraction from the play log data is required to predict whether a user will churn, because not all of the original attributes are directly usable or helpful in prediction. Features influence the prediction capability of a model. In addition, features are useful for understanding prediction results better. In this study, we extracted ten common features for the three games and two game-specific features for Game #2 and #3.

As explained in the Data section, the three games have the same attributes, which are unique ID, score, and time in the play log data. From the attributes, we generated ten common features focusing on the user’s play pattern and game scores. Three features, *playCount*, *activeDuration*, and *consecutivePlayRatio*, are extracted from a user’s play pattern. The feature *playCount* (*n*) is the total number of plays of *u* in the *OP*. The feature *activeDuration* is the time difference between the last play and the first play in the *OP*, which is the same as the relative time of the last play *t*_*n*_ in the *OP*. We use *c*_*i*_ to denote the time difference between two adjacent plays for *consecutivePlayRatio*. That is,

$$c_{i}=t_{i+1}-t_{i},\;where\;i=1,\ldots ,\;n-1.$$

For example, if the second play time of *u* is 1,469,632,182, and the third is 1,469,633,682, then *c*_2_ is 1,500 seconds. The other features are score-related features extracted from basic statistics of a user’s play scores and their transformations. The feature *bestScore (μ*) is the best score earned during the *OP*. That is,

$$\mu =max\;s_{i},\;where\;t_{i}<t_{op}.$$

The feature *meanScore ($\bar{s}$*) is the average score during the OP. That is,

$$\bar{s}=\frac{1}{n}\Sigma_{i=1}^{n}s_{i},where\;t_{i}<t_{op}.$$

Table 5 shows the common features of the three games.

**Table 5:** Common features of the three games.

Feature	Description	Formula
*activeDuration*	Time difference between the last play and the first play in *OP*, the same as the relative time of the last play	*t*_*n*_
*bestScore*	Best score earned in *OP*	*μ*
*bestScoreIndex*	Index of the best score normalized by play count	$\frac{1}{n}argmax_{i}s_{i}$,$\;where\;\;t_{i}<t_{op}$
*bestSubMeanCount*	Difference between best score and mean score normalized by play count	$\frac{\left(\mu -\bar{s}\right)}{n}$
*bestSubMeanRatio*	Ratio between the best score minus mean score and the mean score	$\frac{\left(\mu -\bar{s}\right)}{\bar{s}}$
*consecutivePlayRatio*	Ratio between the count of consecutive plays, where *c*_*i*_ is less than C, and the total number of adjacent plays (C: a game specific duration threshold to distinguish whether two adjacent plays are consecutive plays or not)(	$\frac{count\;of\;c_{i}}{n-1},\;where\;c_{i}<C\;and\;t_{i}<t_{op}$
*meanScore*	Mean score earned in *OP*	$\bar{s}$
*playCount*	Total number of plays of *u* in *OP*	*n*
*sdScore*	Standard deviation (SD) of the scores in *OP*	$\sqrt{\frac{1}{n-1}\sum_{i=1}^{n}{\left(s_{i}-\bar{s}\right)}^{2}},\;where\;\;t_{i}<t_{op}$
*worstScore*	Worst score earned in *OP*	*min s*_*i*,_ *where t*_*i*_ <*t*_*op*_

Game-specific features are extracted from the data fields that are related to genre or characteristics of the game. For instance, since Game #2 is a racing game, vehicle can be upgraded in a store and the purchase related information is reflected in the play log data. Similarly, since Game #3 is a team-play game where a team can win or lose, counts of winning and losing can be derived from play log data. In addition, Game #3 provides play duration information for each game, which is not available for Game #1 and #2.

To introduce game-specific features, we adopt a few extra notations. For Game #2, we use *p*_*j*_ to represent the price paid for the *j*^*th*^ vehicle, where *j = 1*, *2*, *…*, *L*. We let $t_{j}^{pur}$ represent the relative time of the *j*^*th*^ purchase. For Game #3, whether the player wins the *i*^*th*^ play or not is encoded in binary. That is,

$$w_{i}=\{\begin{array}{ll} 1, & if\;points\;of\;i_{\;}^{th}game>0\\ 0, & if\;points\;of\;\;i_{\;}^{th}\;game=0 \end{array}.$$

We let *d*_*i*_ represent the playing duration of the *i*^*th*^ play (*ending time—starting time of each play)*. Table 6 shows game-specific features for Games #2 and #3.

**Table 6:** Game-specific features of Game #2 and Game #3.

Game	Feature	Description	Formula
**Game #2**	*highestPrice*	Highest price among vehicle purchases in *OP*	$max\;p_{j},\;where\;t_{j}^{pur}<t_{op}$
	*purchaseCount*	Total count of vehicle purchases in *OP*	$j,\;where\;t_{j}^{pur}<t_{op}$
**Game #3**	*meanGameDuration*	Mean play duration in *OP*	$\frac{1}{n}\sum_{i=1}^{n}d_{i},\;where\;t_{i}<t_{op}$
	*winRatio*	Ratio between the total number of winning games and the total number of games	$\frac{1}{n}\sum_{i=1}^{n}w_{i},\;where\;t_{i}<t_{op}$

### Single feature ranking

Among the features that were extracted, some are more important than the others in terms of their contributions to churn prediction. To understand which features are the most important ones for each game, we perform a single feature ranking analysis by adopting the variable ranking strategies described in Guyon(2003) [32]. First, we evaluate rankings in five different ways by examining five different scores—absolute value of Pearson correlation coefficient between a feature and churn, three classification performance values (AUC) of the three traditional machine algorithms using only a single feature as the input, and the feature importance that is calculated by gradient boosting library. Then, the overall ranking is determined by comparing the average ranking of the five results.

**Fig 3:**
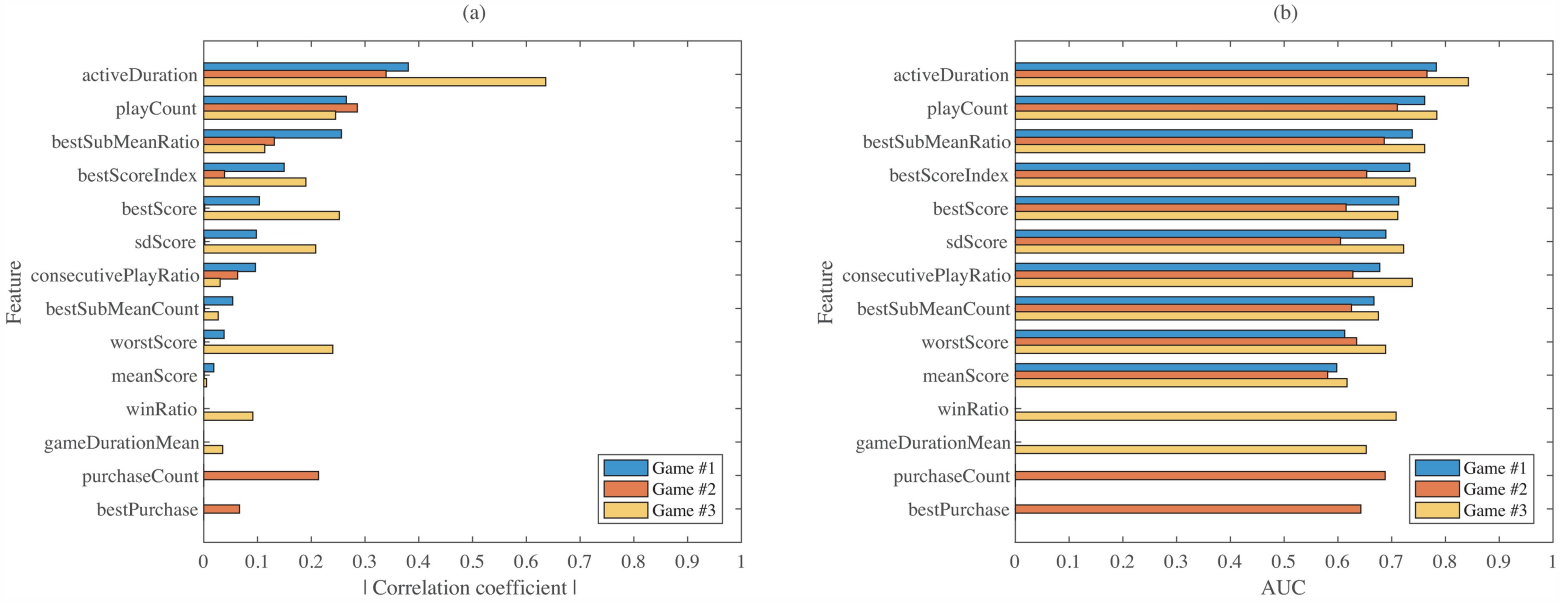
Evaluation of single features. (a) Based on correlation with churn. (b) Based on gradient boosting performance.

Among the five, two of them are shown in Fig 3. Correlation values are shown in (a), and gradient boosting’s AUC values are shown in (b). In both figures, the bottom four features are the game-specific features. For correlation in (a), it can be observed that the highest value occurs for Game #3 when the feature is *activeDuration*. On the other hand, *meanScore* turns out to be the worst feature. Similar results can be found in (b) in terms of ranking. Overall single feature rankings, by averaging the five, are shown in Table 7. For all three games, *activeDuration* and *playCount* are ranked as the top two important features. This implies that play-time related metrics matter much more than play-score related metrics for casual games. For Game #2, purchase related features, that are game-specific features available only for Game #2, rank as the next two important features. For Game #1, *bestScore* ranks at the third position. For Game #3, however, *worstScore* ranks at the third position. While the best score is an important metric for Game #1 that is the simplest among the three games, the worst score is an important metric for Game #3 that is a team-play game.

**Table 7:** Overall average rankings for single features.

Rank	Game #1	Game #2	Game #3
**1**	*activeDuration*	*activeDuration*	*activeDuration*
**2**	*playCount*	*playCount*	*playCount*
**3**	*bestScore*, *consecutivePlayRatio*	*purchaseCount*	*worstScore*
**4**		*bestPurchase*, *consecutivePlayRatio*	*bestScoreIndex*
**5**	*sdScore*		*winRatio*
**6**	*bestScoreIndex*	*bestSubMeanRatio*	*bestSubMeanRatio*
**7**	*bestSubMeanRatio*	*worstScore*	*bestScore*
**8**	*bestSubMeanCount*	*bestScore*	*consecutivePlayRatio*, *sdScore*
**9**	*worstScore*	*bestScoreIndex*	
**10**	*meanScore*	*bestSubMeanCount*	*bestSubMeanCount*, *gameDurationMean*
**11**	-	*sdScore*	
**12**	-	*meanScore*	*meanScore*

### Feature combinations

While single feature analysis provides useful insights on the common traits and the individual characteristics of the three casual games, ultimately multiple features need to be used for the best performance of churn prediction. When multiple features are used, a feature may contain redundant information or noise-dominated information, and therefore feature selection needs to be applied. Saeys (2007) provides a succinct summary where feature selection methods are categorized into filter, wrapper, and embedded searches [31]. While a variety of techniques are available, we choose the most brute-force method of checking performance of all possible feature combinations and identifying the best. This full search is possible because we have only 10~12 features to consider for each game. To simplify the analysis, only gradient boosting algorithm is used for the evaluations.

The results are shown in Table 8. For Game #1, nine out of ten features are used for the best performance. The missing one is *worstScore*, and possibly the worst score is random and conveys no information because many players freely stop playing when interrupted in real-life. For Game #2, four features are used and a game-specific feature, *bestPurchase*, is included. For Game #3, six features are used and a game-specific feature, *winRatio*, is included. For Game #3, it can be also observed that *worstScore* is not included in the best combination, even though it ranked number three in single feature ranking. One can infer that the feature’s churn-related information is mostly redundant when other features are present.

**Table 8:** Feature combination result. The underlined features are the top 2 from the single-feature ranking.

Game	Number of Features	Feature Combination	AUC
**Game #1**	9	*activeDuration*, *bestScore*, *bestScoreIndex*, *bestSubMeanCount*, *bestSubMeanRatio*, *consecutivePlayRatio*, *meanScore*, *playCount*, *sdScore*	0.793
**Game #2**	4	*activeDuration*, *bestPurchase*, *consecutivePlayRatio*, *playCount*	0.720
**Game #3**	6	*activeDuration*, *consecutivePlayRatio*, *meanScore*, *playCount*, *sdScore*, *winRatio*	0.827

**Fig 4:**
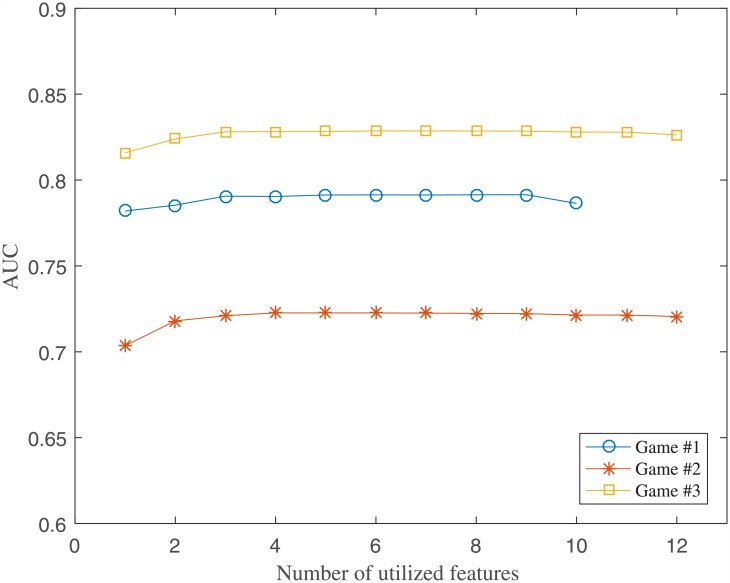
AUC vs. number of utilized features. Increasing number of features has only a marginal effect on AUC performance, especially after 2~3 features are included.

Overall, *activeDuration* and *playCount*, which are the first- and second-ranked features in single-feature ranking, are included in all three games’ combinations (underlined in Table 8). Furthermore, some of the low-ranking features can be found in the combination list, too. Overall, the combination list provides interesting results, but the value of additional features is questionable. In Fig 4, AUC performance as the number of utilized features is shown for all three games. The leftmost point of curve corresponds to the best single-feature performance. It can be seen that the performance improves when the number of features is increased from one to 2~3. Beyond that point, having more features have very marginal effect and the curves look almost flat. Although the best combination results in Table 8 are achieved for 4~9 features, the curves in Fig 4 indicate that casual games are very simple and analysis beyond single-feature ranking might not be very helpful. For the technical completeness, however, the combination lists shown in Table 8 are used for the performance evaluations in the rest of this paper.

## Performance results

Churn prediction performance can be affected by the choice of machine learning algorithm and the choice of *OP* and *CP* in the churn definition. In this section, we investigate the effects of algorithm choice and *OP*/*CP* choice on the AUC performance.

### Performance for different algorithms

We use three traditional algorithms and two deep learning algorithms to investigate performance sensitivity to different machine learning algorithms. To be specific, logistic regression, gradient boosting, and random forests, which are widely used for binary classification problems, are used as the traditional algorithms. A CNN model [34] and a LSTM model [35] are tuned and evaluated as deep learning algorithms. Logistic regression is a linear model for providing the posterior probabilities of multiple classes [36][37]. In contrast, gradient boosting is an ensemble algorithm that increases its performance through randomized subsampling of training data [38]. Random forests is also an ensemble algorithm, and performance is improved by independently generating sampled trees and combining some of the tree predictors [39]. For the implementations of the three algorithms, we used R package’s *xgboost*, *glm*, and *cforest*, respectively [40][41][42].

For research problems such as image classification and natural language processing, deep learning has achieved remarkably enhanced performance in the past decade [29]. One main advantage of deep learning is that it avoids hand-crafting of features (i.e. feature engineering), which is typically required for traditional machine learning algorithms to work well. For image classification, for instance, previously human-coded programs had to be used to detect edges and textures. But, now CNN can automatically extract the edges and textures without human interventions [27]. Here, performance results of deep learning algorithms are provided to investigate if the churn prediction problem of casual games can significantly benefit from automatic feature extractions. Two popular deep learning algorithms are evaluated. For CNN, we use two convolutional layers with successive pooling layers followed by two fully connected layers. We tuned the hyper-parameters such as numbers of filters, filter sizes, and pooling sizes using a grid search. For LSTM, we tuned the number of neurons and input dimensions using a grid search. The deep learning algorithms were implemented using TensorFlow 1.0 library.

**Fig 5:**
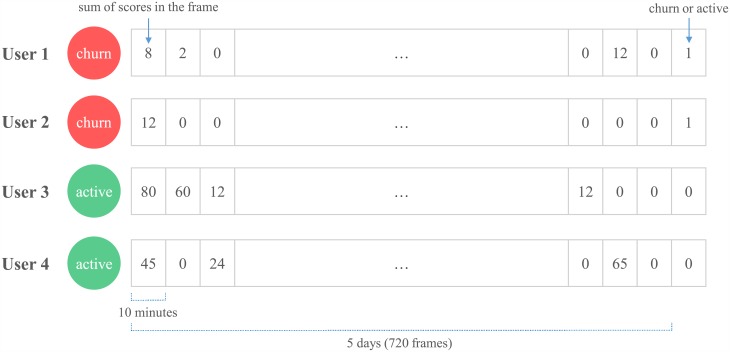
Input to the deep learning algorithms. For each user, the value in each time frame is the sum of all scores that were recorded during the time frame.

For the three traditional algorithms, the carefully designed features from the feature engineering section are used as the inputs. For deep learning algorithms, however, we do not use the extracted features. Instead, the raw data of three games are minimally processed such that the data’s time patterns potentially can be utilized by the deep learning algorithms. To be precise, play log data is transformed into a one-dimensional vector that can be used as the input to the deep learning algorithms. For the transformation, a time framing was adopted as shown in Fig 5. In the figure, the value for each 10-minute time frame is calculated as the sum of all available play scores whose play end-time falls into the time frame. The aggregation of the scores in each time frame causes a slight reduction in information resolution, but the effect should be minimal because the size of a time frame is only 10 minutes.

Table 9 summarizes the performance results of the five algorithms. A rigorous evaluation was performed by first setting aside 20% of the data as test data, and using the other 80% for 10-fold cross-validation. We allocated an independent test data set, as opposed to reusing validation data as test data, because training plus validation data was used for tuning hyper-parameters of traditional machine learning algorithms and deep learning algorithms, and because the evaluation accuracy can be compromised unless an independent test data set is used for the final performance calculation. LSTM performed the best for Game #1, gradient boosting for Game #2, and both of logistic regression and gradient boosting for Game #3. While the winner varied over the three games, the performance advantage of the best performing algorithm was not very large compared to any other algorithm. This indicates that the data of the three casual games might not have a strong structure or pattern to take advantage of, and therefore simply running a reasonably well chosen machine learning algorithm might suffice for churn prediction. Also, it can be observed that the performance of deep learning algorithms is comparable to the performance of traditional algorithms. In fact, deep learning algorithms performed slightly worse for Game #2 and Game #3. This indicates that the play patterns in time dimension is not very helpful for predicting churn, and simply utilizing *activeDuration* and *playCount* can be sufficient.

**Table 9:** Performance (AUC) comparison of three conventional algorithms and two deep learning algorithms (*OP* = 5 days, *CP* = 10 days).

Game	Logistic regression	Gradient boosting	Random forests	CNN	LSTM
**Game #1**	0.786	0.791	0.787	0.774	**0.792**
**Game #2**	0.711	**0.728**	0.722	0.691	0.686
**Game #3**	**0.824**	**0.824**	0.822	0.798	0.803

Only AUC results are shown in Table 9. The best accuracy values can be found by tuning the thresholds, and for gradient boosting they turn out to be 0.93, 0.86, and 0.85 for Game #1, #2, and #3, respectively. The values are high, but the high accuracy is simply because of many players quickly becoming churners for casual games. In general, AUC is a better evaluation metric, because it does not depend on a threshold [43].

### Performance for different choices of *OP* and *CP*

All analysis results so far are based on *OP* = 5 days and *CP* = 10 days. While the choice of algorithm does not affect AUC much, the choice of *OP* and *CP* can have a substantial impact. To investigate the sensitivity, we have evaluated AUC for *OP* between 1 and 30 days and *CP* between 1 and 30 days. For Game #2, an exception was made and *OP* was evaluated only up to 15 days because the data collection period was relatively shorter (3.5 months) and buffer periods were set in the beginning and ending part of the data collection periods to prevent boundary effects. A total of 900 combinations (450 for Game #2) were evaluated for the three casual games, where the overall best performing gradient boosting algorithm was used with ten-fold cross-validation.

**Fig 6:**
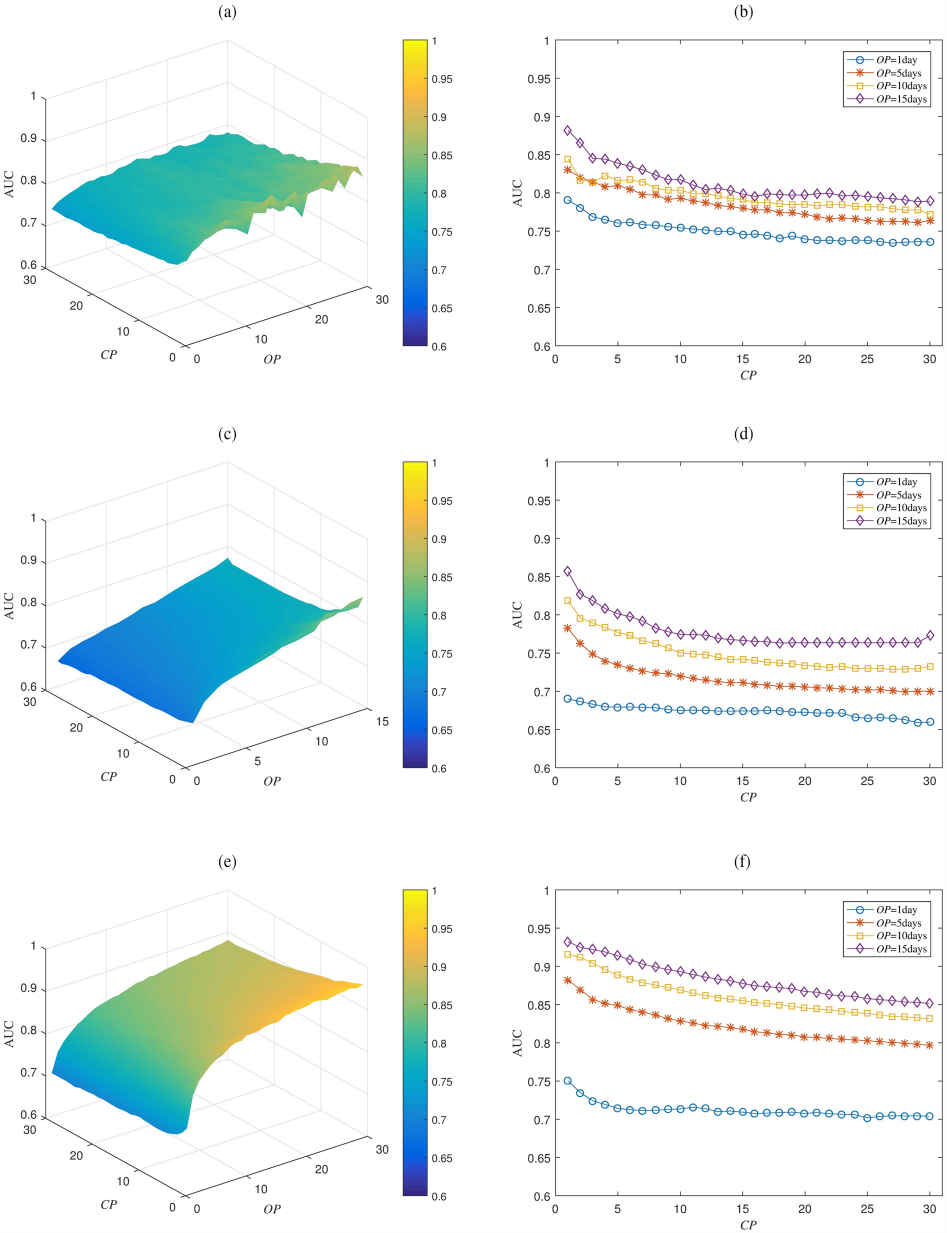
AUC performance as a function of *OP* and *CP* selections. The churn prediction performance is improved by increasing number of observation days and by decreasing number of churn prediction days. (a)(c)(e) AUC vs. (*OP*,*CP*) for Game #1, #2, and #3, respectively (b)(d)(f) AUC vs. *CP* for four fixed *OP* values for Game #1, #2, and #3, respectively.

The results are shown in Fig 6, where three dimensional plots along with two dimensional plots are shown for Game #1, #2, and #3. In the case of *OP*, increasing *OP* has a strongly positive effect on the prediction performance. By having a longer observation period, more data for playing pattern and playing frequency can be obtained. Furthermore, churn players typically start showing less frequent play history as time goes by, and it becomes easier to distinguish typical churners if we wait longer. Choosing a large *OP*, however, has a few downsides as well. First, a large *OP* means a longer waiting time before churn can be predicted. If *OP* = 30 days, for instance, it means we can predict churn rate of the players who have started playing at least 30 days ago, and 30 days can be an unacceptably long time when a newly released casual games need to be quickly evaluated. Secondly, if we wait long enough, there might be almost no chance to contact the ‘likely-to-churn’ players and take preventive actions. For mobile games, preventive actions should be taken at least before the app is removed from the device.

In the case of *CP*, the effect is reversed and decreasing *CP* has a positive effect on prediction performance. By having a shorter churn prediction period, the prediction algorithm can focus on the short-term behaviors without worrying about long-term churning or returning. The effect is easily noticeable for *CP* between 1 and 5 days. The influence, however, becomes weaker or even unnoticeable after 5 days. As explained before, this implies that even *CP* = 5 days or larger might be sufficient to model most players’ long-term behaviors as well as short-term behaviors for casual games. Overall, there are tradeoffs for choosing *OP* and *CP*, and they are considered further in the discussion section.

## Discussions

The behaviors of game players cannot be the same as the behaviors of the telecom or newspaper subscribers. Moreover, behaviors of casual game players can be even more different because of the light and temporal nature of playing casual games. People play for fun and easily quit without worrying about a contract or a subscription fee. By deep diving into the play log data of three casual games, we revealed the key insights that characterize casual games. The results are discussed further in this section.

The first result concerns single-feature analysis and feature combinations. We analyzed each single feature to determine its contribution to churn prediction performance. We defined 14 features, created five rankings, and then summarized the overall ranking by normalizing and averaging the five rankings. For all three games, *activeDuration* and *playCount* features took first and second positions. Not surprisingly, this indicates that a shorter playing time and a lower play count are more relevant to churning. On the other hand, *meanScore* feature took the lowest rank for all three games. One possible explanation is that casual game players often become distracted and quit in the middle of the play and therefore *meanScore* does not carry much of a meaning. Another possible explanation is that individual’s skills and attitudes are vastly diverse that *meanScore* is difficult to utilize for characterizing individual players. Interestingly, *bestScore* and *worstScore* ranked 3^rd^ for Game #1 and Game #3, respectively. Game #1 happens to be the simplest game of the three, and Game #3 happens to be a team-play game where players might be keen to team members’ assessments. Game-specific features, such as *purchaseCount* and *winRatio*, ranked 3^rd^ and 5^th^, respectively for Game #2 and Game #3. They reflect the essential factors of each game’s design, but ended up being less relevant than *activeDuration* and *playCount*. As for the feature combination analysis, the most important insight is that only 2~3 high-ranking features are needed for churn prediction. Technically, the best performance is obtained by using 4~9 features, but the performance improvement is negligible after using 2~3 features. We believe this is due to lack of complexity and diversity in the way casual game players behave. Casual games are quite different from other traditional games, and probably there are only a small number of factors that drive a player’s behavior and the resulting churn. In this case, it is very important to choose effective features for monitoring users and making action decisions, but only a handful of well-crafted and validated features may be used safely for fully understanding user behavior.

The second result concerns the comparison of prediction performance of the five algorithms. LSTM and gradient boosting algorithms had the best prediction performance for the three games, but the performance advantage over the worst performance was small (AUC difference of 0.042 or less). As for the two deep learning algorithms, both of CNN and LSTM performed about same or slightly worse than the three traditional machine learning algorithms. This indicates that the deep learning’s inherent capability to build and use features automatically by utilizing time-dimension patterns was not very helpful. Even though not included in this paper, additional studies including frame size tuning of both CNN and LSTM were conducted, but there was no significant improvement. The play log data of casual games is relatively simple, and our results indicate that indeed the prediction performance has little dependency on the choice of machine learning algorithm.

The last result concerns the definition of churn by choosing *OP* and *CP*. For churn analysis of non-subscription services, the performance can drastically change depending on how churn is defined. Therefore, it is essential to understand the tradeoffs and to specify the details. For the three casual games that we have analyzed, AUC performance can widely vary from below 0.7 to above 0.9 depending on the choice of *OP* and *CP*. Increasing observation period and decreasing churn prediction period can be helpful for enhancing performance value, but there are tradeoffs with how long one needs to wait before churn rate can be predicted and if the performance value well represents the business aspects that one is interested in. For casual games, a few to several days seem to be appropriate for both OP and CP, and we have used 5 and 10 days for most of our analyses.

A limitation of this research lies in the datasets of the three games. Casual games are becoming less simple as time goes by, and social functions (such as chatting, friend list, and team plays) and other new functions are being adopted. We have examined features relevant to item purchase (Game #2) and team winning (Game #3) in this study, but they are not sufficient for understanding future casual games, and further studies are needed.

## Conclusions

In this work, we analyzed three casual games by performing churn predictions. Through the analysis, we demonstrated how play log data can be used to conduct a fundamental set of churn analyses. Churn was carefully defined for casual games, and a standard process for analysis was established. The definition and process are directly applicable not only to casual games, but also to any new user analysis of non-subscription-based services. Overall, the main findings in this study are due to the *simple* nature of casual games. If the findings generally hold true for most of casual games, choosing an appropriate churn definition and identifying a few core features can be the most important parts of understanding and preventing churn.

## References

[1] Egenfeldt-Nielsen S, Smith JH, Tosca SP. Understanding video games: The essential introduction: Routledge; 2016.

[2] Juul J. A casual revolution: Reinventing video games and their players: MIT press; 2010.

[3] Orji R, Vassileva J, Mandryk RL. LunchTime: a slow-casual game for long-term dietary behavior change. Personal and Ubiquitous Computing. 2013;17(6):1211–21.

[4] Kultima A, editor Casual game design values. Proceedings of the 13th international MindTrek conference: Everyday life in the ubiquitous era; 2009: ACM.

[5] Kuittinen J, Kultima A, Niemelä J, Paavilainen J, editors. Casual games discussion. Proceedings of the 2007 conference on Future Play; 2007: ACM.

[6] Association ES. Essential facts about the computer and video game industry. 2010.

[7] Koetsier J. Mobile app monetization: Freemium is king, but in-app ads are growing fast. VentureBeat, 3 2014.

[8] Reichheld FF, Schefter P. E-loyalty: your secret weapon on the web. Harvard business review. 2000;78(4):105–13.

[9] Ganesh J, Arnold MJ, Reynolds KE. Understanding the customer base of service providers: an examination of the differences between switchers and stayers. Journal of marketing. 2000;64(3):65–87.

[10] Au W-H, Chan KC, Yao X. A novel evolutionary data mining algorithm with applications to churn prediction. IEEE transactions on evolutionary computation. 2003;7(6):532–45.

[11] Hadiji F, Sifa R, Drachen A, Thurau C, Kersting K, Bauckhage C, editors. Predicting player churn in the wild. 2014 IEEE Conference on Computational Intelligence and Games; 2014: IEEE.

[12] Kwon H, Mohaisen A, Woo J, Kim H, Kim Y, Lee E. Crime Scene Reconstruction: Online Gold Farming Network Analysis. IEEE Transactions on Information Forensics and Security. 2017;12(3):544–556.

[13] Lee E, Woo J, Kim H, Mohaisen A, Kim H. You Are a Game Bot!: Uncovering Game Bots in MMORPGs via Self-similarity in the Wild. Proc. Netw. Distrib. Syst. Secur. Symp.(NDSS); 2016.

[14] Woo J, Choi H, Kim H. An automatic and proactive identity theft detection model in MMORPGs. Applied Mathematics & Information Sciences. 2012;6(3):291.

[15] Kwak H, Blackburn J, Han S. Exploring Cyberbullying and Other Toxic Behavior in Team Competition Online Games. Proceedings of the 33rd Annual ACM Conference on Human Factors in Computing Systems; 2015: ACM.

[16] Kwak H, Blackburn J. Linguistic Analysis of Toxic Behavior in an Online Video Game. International Conference on Social Informatics; 2014: Springer.

[17] Hanley JA, McNeil BJ. The meaning and use of the area under a receiver operating characteristic (ROC) curve. Radiology. 1982;143(1):29–36. doi: 10.1148/radiology.143.1.7063747 7063747 10.1148/radiology.143.1.7063747

[18] Bamber D. The area above the ordinal dominance graph and the area below the receiver operating characteristic graph. Journal of mathematical psychology. 1975;12(4):387–415.

[19] Kohavi R, editor A study of cross-validation and bootstrap for accuracy estimation and model selection. Ijcai; 1995.

[20] Coussement K, Van den Poel D. Churn prediction in subscription services: An application of support vector machines while comparing two parameter-selection techniques. Expert systems with applications. 2008;34(1):313–27.

[21] Xie Y, Li X, Ngai E, Ying W. Customer churn prediction using improved balanced random forests. Expert Systems with Applications. 2009;36(3):5445–9.

[22] D Anil Kumar, V Ravi. Predicting credit card customer churn in banks using data mining. International Journal of Data Analysis Techniques and Strategies. 2008;1(1):4–28.

[23] Morik K, Köpcke H, editors. Analysing customer churn in insurance data–a case study. European Conference on Principles of Data Mining and Knowledge Discovery; 2004: Springer.

[24] Runge J, Gao P, Garcin F, Faltings B, editors. Churn prediction for high-value players in casual social games. 2014 IEEE Conference on Computational Intelligence and Games; 2014: IEEE.

[25] Kawale J, Pal A, Srivastava J, editors. Churn prediction in MMORPGs: A social influence based approach. Computational Science and Engineering, 2009 CSE'09 International Conference on; 2009: IEEE.

[26] Hinton GE, Salakhutdinov RR. Reducing the dimensionality of data with neural networks. Science. 2006;313(5786):504–7. doi: 10.1126/science.1127647 16873662 10.1126/science.1127647

[27] LeCun Y, Bengio Y, Hinton G. Deep learning. Nature. 2015;521(7553):436–44. doi: 10.1038/nature14539 26017442 10.1038/nature14539

[28] Szegedy C, Liu W, Jia Y, Sermanet P, Reed S, Anguelov D, et al., editors. Going deeper with convolutions. Proceedings of the IEEE Conference on Computer Vision and Pattern Recognition; 2015.

[29] Wangperawong A, Brun C, Laudy O, Pavasuthipaisit R. Churn analysis using deep convolutional neural networks and autoencoders. arXiv preprint arXiv:160405377. 2016.

[30] Chandrashekar G, Sahin F. A survey on feature selection methods. Computers & Electrical Engineering. 2014;40(1):16–28.

[31] Saeys Y, Inza I, Larrañaga P. A review of feature selection techniques in bioinformatics. bioinformatics. 2007;23(19):2507–17. doi: 10.1093/bioinformatics/btm344 17720704 10.1093/bioinformatics/btm344

[32] Guyon I, Elisseeff A. An introduction to variable and feature selection. Journal of machine learning research. 2003;3(Mar):1157–82.

[33] Coussement K, Benoit DF, Van den Poel D. Improved marketing decision making in a customer churn prediction context using generalized additive models. Expert Systems with Applications. 2010;37(3):2132–43.

[34] Krizhevsky A, Sutskever I, Hinton GE, editors. Imagenet classification with deep convolutional neural networks. Advances in neural information processing systems; 2012.

[35] Hochreiter S, Schmidhuber J. Long short-term memory. Neural computation. 1997;9(8):1735–80 9377276 10.1162/neco.1997.9.8.1735

[36] Friedman J, Hastie T, Tibshirani R. The elements of statistical learning: Springer series in statistics Springer, Berlin; 2001.

[37] Murphy KP. Machine learning: a probabilistic perspective: MIT press; 2012.

[38] Friedman JH. Stochastic gradient boosting. Computational Statistics & Data Analysis. 2002;38(4):367–78.

[39] Breiman L. Random forests. Machine learning. 2001;45(1):5–32.

[40] Nelder JA, Baker RJ. Generalized linear models. Encyclopedia of statistical sciences. 1972.

[41] Chen T, He T. xgboost: eXtreme Gradient Boosting. R package version 04–2. 2015.

[42] Chen T, Guestrin C. Xgboost: A scalable tree boosting system. arXiv preprint arXiv:160302754. 2016.

[43] Burez J, Van den Poel D. Handling class imbalance in customer churn prediction. Expert Systems with Applications. 2009;36(3):4626–36.

